# Future research directions to identify risks and mitigation strategies for neurostructural, ocular, and behavioral changes induced by human spaceflight: A NASA-ESA expert group consensus report

**DOI:** 10.3389/fncir.2022.876789

**Published:** 2022-08-04

**Authors:** Rachael D. Seidler, Claudia Stern, Mathias Basner, Alexander C. Stahn, Floris L. Wuyts, Peter zu Eulenburg

**Affiliations:** ^1^Department of Applied Physiology & Kinesiology, Health and Human Performance, University of Florida, Gainesville, FL, United States; ^2^Department of Clinical Aerospace Medicine, German Aerospace Center (DLR) and ISS Operations and Astronauts Group, European Astronaut Centre, European Space Agency (ESA), Cologne, Germany; ^3^Department of Psychiatry, Perelman School of Medicine, University of Pennsylvania, Philadelphia, PA, United States; ^4^Department of Physics, University of Antwerp, Antwerp, Belgium; ^5^Laboratory for Equilibrium Investigations and Aerospace (LEIA), Antwerp, Belgium; ^6^German Vertigo and Balance Center, University Hospital, Ludwig-Maximilians-Universität München (LMU), Munich, Germany

**Keywords:** brain, eye, SANS, mitigation, strategies, astronaut, human spaceflight, expert opinion

## Abstract

A team of experts on the effects of the spaceflight environment on the brain and eye (SANS: Spaceflight-Associated Neuro-ocular Syndrome) was convened by NASA and ESA to (1) review spaceflight-associated structural and functional changes of the human brain and eye, and any interactions between the two; and (2) identify critical future research directions in this area to help characterize the risk and identify possible countermeasures and strategies to mitigate the spaceflight-induced brain and eye alterations. The experts identified 14 critical future research directions that would substantially advance our knowledge of the effects of spending prolonged periods of time in the spaceflight environment on SANS, as well as brain structure and function. They used a paired comparison approach to rank the relative importance of these 14 recommendations, which are discussed in detail in the main report and are summarized briefly below.

## Introduction

This document is the result of discussions and work by the NASA-ESA Brain and SANS (Spaceflight-Associated Neuro-ocular Syndrome) Expert Team, which was convened in 2020–2022 by NASA and ESA. The team members were Mathias Basner, Rachael D. Seidler, Alexander C. Stahn, Claudia Stern, Floris L. Wuyts, and Peter zu Eulenburg. The group was charged with (1) reviewing what is known regarding human brain changes occurring with spaceflight, SANS, and any interactions between the two; and (2) identifying critical directions for future research in this area to solve operational and health issues in space crew.

In the past 6 years, substantial evidence has accumulated that spaceflight affects human brain structure (Koppelmans et al., 2016; Roberts et al., 2017; Van Ombergen et al., 2018, 2019; Lee et al., 2019b; Riascos et al., 2019; Hupfeld et al., 2020, 2022; Jillings et al., 2020; Kramer et al., 2020; Roy-O’Reilly et al., 2021; Barisano et al., 2022; Doroshin et al., 2022). These changes include an upward shift of the brain within the cranial compartment, ventricular expansion, perivascular space changes and white matter alterations. A few studies have further shown that these brain changes correlate with changes in functional behaviors such as posture control (Lee et al., 2019b; Roberts et al., 2019; Koppelmans et al., 2022). The nature of these associations supports the interpretation that some brain changes with spaceflight are “dysfunctional”—that is, they are correlated with poorer performance—whereas others appear to reflect adaptive plasticity (Demertzi et al., 2016; Pechenkova et al., 2019; Hupfeld et al., 2021).

Over the past decade, reports of ocular structural and functional changes following spaceflight have also emerged. For example, in 2011, the first seven cases of eye changes in astronauts after 6-month space missions were described (Mader et al., 2011). These changes were initially termed Visual Impairment and Intracranial Pressure (VIIP) syndrome and later renamed Spaceflight Associated Neuro-ocular Syndrome (SANS) to stress the central nervous system (CNS) involvement. Optic disc edema, globe flattening, choroidal and retinal folds, hyperopic refractive error shift, optic nerve sheath distension and areas of ischemic retina (i.e., cotton wool spots) are all part of SANS (Lee et al., 2020). Because of the increasing number of these changes, a survey study of astronauts was conducted by NASA. 29.3% of Space Shuttle astronauts and 47.7% of 6-month International Space Station (ISS) astronauts reported a change of near and/or distance vision (Mader et al., 2011).

Numerous questions remain about the brain and ocular structural changes that occur with spaceflight, including whether and how they relate to each other, what the behavioral consequences of each might be, whether they occur with greater frequency and/or magnitude with longer duration missions, what the characteristics of the postflight recovery timeline are, and what the underlying causal factors and mechanisms may be, to name a few. Here, we lay out our recommendations for the most critical near-term research to protect the health and operational performance of future human space travelers.

We are well aware of constraints in crew time for participating in research, and our recommendations take these constraints into account. We do, however, want to stress that in light of small subject samples repeated assessments can help to better characterize the individual astronaut as well as improve the resolution of temporal changes during all phases of a spaceflight mission.

We acknowledge that the impact of radiation in combination with microgravity is still to be unraveled. All the acquired data to date from low-earth orbit (under the protective umbrella of the magnetosphere) shows no radiation effects on the most susceptible cranial structures, the centrum semiovale of the white matter of the brain. We believe that the impact of radiation in combination with microgravity is still to be unraveled, but beyond the scope of this position paper as no data exist from beyond low-earth orbit. The longitudinal study by Chylack et al. (2012) and Özelbaykal et al. (2022) could not demonstrate a significant relationship between space radiation exposure and progression of aggregate area of posterior subcapsular cataract or nuclear progression rates. There is currently no evidence regarding whether or not radiation interacts with SANS. This said, an increasing number of studies suggest that chronic low-dose radiation effects are expected to be a critical risk factor for brain plasticity and cognitive performance during future exploratory class missions (Cucinotta et al., 2014; Jandial et al., 2018; Acharya et al., 2019; Zwart et al., 2021). Hence, the experts think this is an important topic for future research even though it is not addressed explicitly here.

**FIGURE 1:**
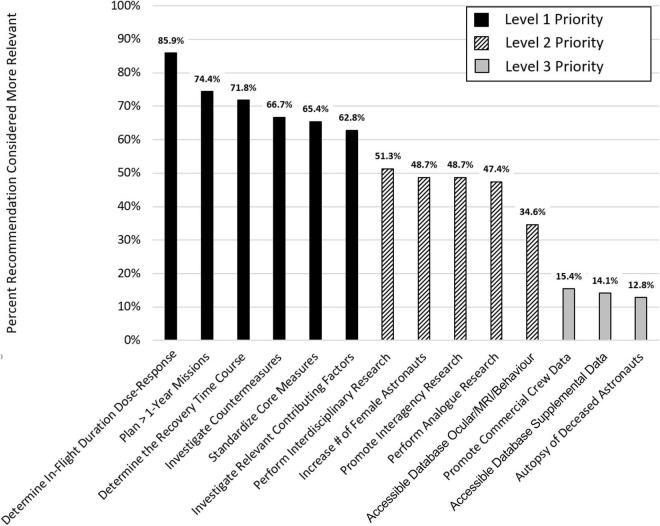
Prioritization of recommendations by the team of experts.

The expert panel recommends 14 measures that would substantially advance our knowledge of the effects of spending prolonged periods of time in the spaceflight environment on SANS as well as brain structure and function. These recommendations are discussed in detail below. The panelists developed the recommendations based on their review of the literature and their working knowledge of the subject. To determine the relevance of each of the 14 recommendations, the expert panel used a paired comparison (PC) approach (Kendall and Smith, 1940). In a PC, two items are contrasted, and a scorer decides which of the two items is more relevant. Fourteen items give rise to 91 possible pairwise recommendation combinations; these were scored individually by each panel member (6 × 91 = 546 total paired comparisons; the order in which pairs were presented was randomized for each rater, and each recommendation appeared in 40–60% as the first item). The results of the PC effort based on the panelists’ pairwise rankings are illustrated in Figure 1. “Determining in-flight duration dose-response relationships” had the highest priority (more relevant in 85.9% of paired comparisons) while “autopsy of deceased astronauts” had the lowest priority (more relevant in only 12.8% of paired comparisons). Based on the PC approach, we identified three clusters of recommendations that were subsequently classified as level 1, level 2, and level 3 priority (see Figure 1) with level 1 being the highest level.

In the recommendations below, whenever we refer to changes in brain and eye, we refer to both structural and functional changes. Functional changes can be manifest as brain activity or behavior; we use the term “brain function” here to broadly refer to both. Moreover, for each of our recommendations, where we outline the need to measure aspects of the brain, eye, and behavior, we recommend that the change-change interrelationships between all three also be evaluated. This will provide insight into potential mechanisms and consequences of change. It is not yet clear whether behavioral changes are related to (or arise from) SANS; given the need for precise operational performance in microgravity this is a critical question. And, there is emerging evidence from bed rest analogs to suggest that signs of SANS are correlated with both brain function and behavior.

## Level 1 priority recommendations

### Recommendation level 1–1: Determine in-flight duration and g-level exposure-response function

Determine the exposure-response relationships for brain and eye changes for mission duration, accumulated days in space, and the effect of g-level transitions for biomarker definition.

Data suggest that the longer a mission takes and the longer a test subject spends in head down tilt during bed-rest studies, the more severe the ocular changes, especially the increase of the retinal nerve fiber layer thickness (Laurie et al., 2019; Macias et al., 2020). Data from shuttle missions show that a 2-week mission duration typically does not induce SANS, whereas a 6-month mission duration does. Hupfeld et al. (2020) also showed greater increases in ventricular volume and free water (cranial fluid) shifts following 1 year in space compared to 6 months. A recent study on the perivascular space in the brain of NASA astronauts showed that shuttle crew had no significant increase after their mission whereas astronauts who spent 6 months in the ISS demonstrated significant perivascular space increase after flight (Barisano et al., 2022). Further, Scott Kelly took significantly longer to re-adapt to Earth’s gravity environment after his 1-year ISS mission compared to his previous 6-month mission (Kelly, 2017; Garrett-Bakelman et al., 2019).

Certain effects build up after 1 to several months in flight, such as increased core temperature, which also persists after landing (Stahn et al., 2017). Ventricular volume increases observed in recent studies also lasted for several months (Van Ombergen et al., 2018, 2019), with larger preflight ventricular volumes in crewmembers who had flown the most recently (Hupfeld et al., 2020). This suggests hysteresis effects, as well as build up or cumulative effects. Case studies showing that astronauts who developed eye changes during the first flight showed even more severe changes during the second flight also support this suggestion (Mader et al., 2013). A recent study reports increased brain perivascular space volume postflight for novice but not experienced astronauts, suggesting that experienced astronauts may exhibit holdover effects from prior spaceflight(s) (Hupfeld et al., 2022). However, another earlier study on perivascular space in a group of over 40 astronauts and cosmonauts, both novice and experienced fliers, showed that both groups developed significant increases in perivascular space after spaceflight. Interestingly, a significantly larger increase in the NASA astronauts was observed compared to Roscosmos cosmonauts (Barisano et al., 2022).

We need to determine exposure-response relationships for adverse brain and eye effects, depending on mission duration, and the effect of accumulated time spent in space. As future missions will involve intermittent periods spent in partial gravity, we also need to investigate which level of gravity is sufficient to mitigate some of the adverse brain and eye effects. These investigations could include sojourns on the Moon surface and/or dedicated (partial) artificial gravity experiments.

### Recommendation level 1–2: Plan missions longer than 1-year

Execute at least a few low Earth orbit missions longer than 1-year to inform structural and functional brain and eye changes induced by prolonged exposure to the spaceflight environment.

As of early April 2022, 583 astronauts participated in 1,314 space missions. More than 50% of these missions were 12 days or shorter, only 8.8% were longer than 180 days and only 4 humans have spent consecutively more than 1 year in space (Smith et al., 2020). Thus, we know very little about how astronaut physiology in general, and the brain and eye more specifically, will be affected if we subject astronauts to the spaceflight environment for more than 1 year. This lack of knowledge is concerning because a mission to Mars and back can take 3 years or longer. Astronauts will be exposed to partial gravity while on the surface of Mars, but we do not know whether it will be sufficient to stimulate partial or full recovery from SANS or other brain changes initiated in microgravity. In addition, other stressors commonly encountered during spaceflight will be more extreme compared to low Earth orbit (LEO) ISS missions. These factors include, but are not limited to, radiation exposure, habitat and crew size, lack of rescue opportunity, and communication delays. For these reasons, it may not be possible to extrapolate the psychological and physiological changes observed during 6–12-month LEO missions to longer mission durations and more extreme environments. The fact that SANS was only identified as a significant problem after a relevant number of astronauts stayed for 6 months aboard the ISS corroborates this concern.

This group therefore suggests investigating at least one male and one female astronaut during and after a stay of up to 2 years on the ISS, currently our best analog for exploration-class space missions, before sending humans to Mars. Due to only partial reversibility of some of the structural brain changes (documented up to R+7 months) induced by spaceflight (Van Ombergen et al., 2019; Hupfeld et al., 2020), the group acknowledges potential ethical issues related to subjecting astronauts to even more extensive periods aboard the ISS. At the same time, questions related to the severity and reversibility of structural and functional brain changes induced by long-duration spaceflight do need to be answered before we can safely send humans to Mars.

### Recommendation level 1–3: Map the recovery time course

Collect core data for brain and eye as close to landing as logistically feasible and at multiple time points up to a minimum of at least 2 years after missions longer than 3 months.

The circulatory and vascular phenomena associated with head and neck venous congestion resulting from cephalad fluid shift in microgravity are probably reversed instantaneously upon re-entry into the normal gravitational environment. Published studies on cerebrospinal fluid (CSF) flow velocities and ventricular expansion used data from 3 to 10 days after landing. Substantial CSF reabsorption and involution of perivascular space expansion will have already occurred at this point, even though enlarged ventricular volumes and gray matter alterations are still measurable up to 6 months postflight (Van Ombergen et al., 2019).

Core clinical neuroimaging data should be acquired as early as logistically feasible. Without neuroimaging and other core data sampling shortly after landing, we run a high risk of underestimating the structural effects on the brain and eye directly related to mission duration and thus being unable to model these effects accurately. Optical coherence tomography (OCT) data, intraocular pressure, and eye ultrasound from the last week in microgravity compared with postflight OCT data would aid in revealing the changes in intracranial pressure (ICP) during the return phase after a long-duration mission. Measuring as close to landing as logistically feasible will help to accurately capture the magnitude of a pre- to post-flight change, allowing us to understand any medical consequences and effects on long-term health. Measurements at these timepoints are also critical for capturing a full recovery trajectory.

SANS features have been shown to persist for more than 2 years after a long-duration mission. Structural changes in gray and white matter of the brain and expanded ventricular volumes have also persisted for at least 6 months after return to Earth (Van Ombergen et al., 2019; Hupfeld et al., 2020). To better delineate tissue normalization processes of the brain from permanent structural effects, a minimum follow-up period of 2 years for brain and eye should become standard protocol. Higher sampling for the initial mission return phase (2–3 months) could be extended to 6- or even 12-month intervals after 1 year.

In some astronauts, the recovery from spaceflight-induced eye changes is very fast; whereas in a few cases, full recovery does not occur, even years after the mission. In test subjects from the 2019 Artificial Gravity Bed Rest ESA (AGBRESA) ground-based, head down tilt bed rest studies, optic disc edema completely reversed within days in some cases. In all test subjects the edema resolved by the 12 months examination after the end of the head down tilt bed rest exposure. Unfortunately, no other analog studies have assessed optic disc edema recovery; so, the number of subjects is still small, and more studies are needed to be conclusive.

### Recommendation level 1–4: Standardize core measures for space and space analog environments

Expand and standardize the core measures in space and space analog research of brain, eye and behavior to allow for cohort and effect size comparisons between environments and experiments.

To fully understand the effects of spaceflight on the human brain and eye, and the resulting impact on behavior, an expanded and standardized set of core measures should be administered internationally. These measures should be harmonized across not only spaceflight, but also analog environments.

The suggested minimal set of standardized core measures are:

#### Ocular measurements

Minimum core ocular measurements should be performed that take all parameters that are changed by microgravity (and its simulations) into account.

Minimum core measurements should be performed in all space analog experiments and during spaceflight. Newer and more advanced space analog equipment needs to be considered to receive more specific information about different eye changes. This additional sophisticated equipment will probably not match the instruments that are on the ISS but will deliver a better understanding of the origin of the syndrome. Data acquisition can be optimized (especially in OCT scans and software evaluation). Core measurements should assess possible eye changes with methods that will be available in most space analogs and be performed *at least* pre- and post-mission (0–3 days after return from space). To follow the potential changes, inflight measurements are also crucial and should be performed at least three times during a 6-month mission or a bed rest study 30 days or longer. For missions longer than 6 months, more data points should be collected to reveal the time course of changes.

• Visual acuity: Reduced visual acuity can impact any mission; therefore, it is important to identify and mitigate negative changes.
• Optical biometry: This is the most exact method to identify a reduced length of the eye, which can occur when the globe flattens and may result in hyperopic shift.
• Intraocular pressure: The relationship of intraocular pressure to CSF circulation in retro-orbital space is important in identifying the underlying mechanism of globe flattening and optic disc edema.
• Ultrasound of globe and optic nerve sheath: This is an easy method to perform and to identify globe flattening and optic nerve sheath distension and will be available also on long-term missions since it can meet size and weight limitations of the spacecraft.
• Fundus: Photography by a retinal fundus camera or MultiColor Module by OCT can indicate retinal changes by revealing any choroidal and retinal folds or ischemia-induced cotton wool spots.
• OCT (see Appendix C for further details): OCT is the most important and accurate method to reveal microscopic changes in the central retina and optic nerve, including comparison measurements of retinal and optic disc thickness.
• Optic Nerve and Brain Imaging via MRI: These data should include high-resolution (minimum 0.5–1 mm, may vary depending on sequence type or brain region of interest) 3D T1-, T2-, and susceptibility-weighted images of the head (see health surveillance neuroimaging section below for more details) with a minimum 6-month interval follow-up for 2 years after any long-duration mission (defined as > than 3 months).

#### Health surveillance neuroimaging

Core health surveillance neuroimaging of the brain and eye should occur as early as logistically possible after mission return (<24 h). Neuroimaging capabilities at sites relied upon by NASA are limited and in need of hardware modernization.

We advise standardized, high-quality, up-to-date MRI protocols for the brain and eye as part of the long-term health surveillance immediately after a space mission (cf. Roberts et al., 2020). Considering the risk for cranial venous congestion as indicated by jugular vein thrombosis, we also advise a dedicated venous vasculature sequence (such as susceptibility-weighted, blood iron-sensitive).

Considerable evidence suggests that the environmental, operational, and psychological stressors associated with prolonged spaceflight can impair hippocampal plasticity and negatively affect performance, and behavior. Radiation (Parihar et al., 2015), CO_2_ levels (Kiray et al., 2014), vestibular dysfunction (Smith, 2017), stress (Fa et al., 2014), sleep deprivation (Raven et al., 2019), and isolation and confinement (Cinini et al., 2014) each also affect brain health (see Appendix B for details). Neuroimaging measurements must be taken immediately (R+0) after mission return (postflight) to fully understand the degree of intracranial fluid accumulation and structural consequences on the brain. A pilot study in blood-based biomarkers was only recently able to delineate some of the structural consequences for the human brain directly after a long-duration mission (Zu et al., 2021). The timely neuroimaging information will also help reveal all potential clinical aspects of altered head circulation in microgravity, including the upstream intracranial risks associated with the possibility of a jugular vein thrombosis. A major reversal of circulatory blood volume changes should be observable within the first hours after landing.

All core MRI sequences should be acquired in isotropic (3D) resolution where feasible for the respective contrast to allow unbiased quantification of all clinical phenomena with respect to SANS and the brain. This resolution will also allow for long-term support and usability of all data. The core sequences can provide a general neurological and ophthalmological health surveillance, while at the same time acting as supplementary core research elements for use at a later point in time (thus reducing overall scanning crew time later). Please see Appendix B for the proposed core health surveillance MRI protocol and data quality control in detail. We recognize that it may be difficult to implement all of our recommended sequences into health surveillance imaging; this effort could also be accomplished with targeted research projects.

The expert team recognizes that these requirements may be hard to meet at older or multiple-machine facilities. The concerns include the current MRI systems available at UTMB Victory Lakes, which are conveniently located in the vicinity of NASA Johnson Space Center and minimize astronaut time required for scans but are outdated. The use of the *same* MRI scanner, however, is important to reliably identify structural and functional brain changes in longitudinal studies. In addition, MRI technologies have significantly advanced during recent years, and now allow more tissue-specific, precise, and time-efficient brain imaging. Given the older age of the present MRI system at UTMB Victory Lakes, some of the latest state-of-the-art imaging protocols for monitoring brain health cannot be implemented. Furthermore, long-term studies exceeding standard ISS mission durations, and studies that follow recovery for several years, may exceed the lifetime of these systems. The expert team acknowledges the criticality of crew time and minimizing travel for scans but suggests that clinicians consider whether an alternate, updated MRI system featuring the latest technologies might be available soon to address the strong need for state-of-the-art brain imaging. This need does not really apply to ESA researchers since the fully equipped dual-use 3T scanner (PET-MRI) at DLR :envihab is mid-to-late term now (7 years old) and represents a good upgradable, long-term solution at least until the end of the lifetime of the ISS. This MRI is a research-only scanner integrated into facilities of the astronaut training and return center which at the same time are home to bed rest studies and a short-arm human centrifuge.

#### Cognition and operational performance measures

To facilitate data pooling and meta-analyses, every astronaut should perform a minimal set of assays that probe cognitive and operational performance. We suggest using NASA’s Cognition and ROBoT-r tasks and to consider using the newly flight-certified Spatial Cognition test battery.

Astronauts have reported cognitive symptoms often referred to as “space fog” or “neurasthenia” especially after initial exposure to the spacecraft environment (Kanas et al., 2001). However, the results of objective cognitive testing in spaceflight vary and often fail to show statistically significant changes (Strangman et al., 2014; Hupfeld et al., 2021). Several factors likely explain the lack of changes in cognitive performance despite numerous insults to the CNS that can affect cognition and are inherent to spaceflight (e.g., microgravity and radiation). These include, but are not limited to: (a) practice effects, i.e., improving performance with repeated administration that may mask spaceflight effects; (b) the lack of adequate ground-based control groups and ground-based normative data for the astronaut population; (c) small number of astronauts and test administrations and the related low statistical power; (d) low sensitivity of cognitive instruments; (e) limited coverage of cognitive domains, i.e., test batteries may be too narrow and fail to detect deficits in domains not assessed by the selected tests; (e) highly trained and motivated astronauts may be able to transiently compensate for spaceflight-induced deficits in cognitive performance; and (f) countermeasures that are in place to mitigate the effects of the spaceflight environment on astronauts that may also benefit cognition (Basner et al., 2015).

Computerized tests typically administered in spaceflight usually assess either basic cognitive functions (e.g., memory, abstraction) or operationally relevant performance (e.g., docking simulators). While performance on basic cognitive and operational tasks are related (Basner et al., 2020b), it is nevertheless important to capture spaceflight-induced deficits separately for both types of task. Cognitive performance has been a focus of many spaceflight investigations; however, each investigator tends to use specific and non-standardized instruments which prevents pooling data across studies and astronauts and systematic meta-analyses (Strangman et al., 2014). Experimenters should instead administer the same set of tests in all astronauts and ground analogs, in addition to tests of specific interest to each investigator. NASA’s standard cognitive test battery Windows Spaceflight Cognitive Assessment Tool (WinSCAT) has been used operationally for many years (Kane et al., 2005). Unfortunately, the five Automated Neuropsychological Assessment Metrics (ANAM) subtests that comprise WinSCAT only cover a limited spectrum of cognitive functions with a focus on working memory.

Our recommendations aim to mitigate some of the shortcomings described above and are in line with decisions made for NASA’s Standard Measures project (Perez, 2018): We recommend using NASA’s Cognition test battery (Basner et al., 2015) and NASA’s Robotics On-Board Trainer (ROBoT-r) (Ivkovic et al., 2019) to assess basic cognitive functions and complex operational performance, respectively. These measures should be assessed in all astronauts and subjects participating in ground analog research studies whenever feasible for comparison. There is also increasing evidence that visuospatial abilities are particularly vulnerable during spaceflight (Reschke and Clément, 2018 for review). Spatial updating, path integration, route learning, way-finding, and cognitive mapping are key to successfully navigating in novel small- and large-scale environments (Wolbers and Wiener, 2014). The criticality of encoding representations about self-to-object relations and integrating this information into a spatial map of the environment for spaceflight operations was highlighted during the Apollo 14 mission (Stahn and Kühn, 2021). Performance monitoring of astronauts should therefore include tasks assessing the encoding, processing, storage, and retrieval of visuospatial information. The recommended profile is thus:

• Cognition—This battery consists of 10 brief cognitive tests specifically designed for high-performing astronauts that cover a range of cognitive domains, including visuospatial and working memory, spatial orientation, abstract reasoning and concept formulation, emotion recognition, abstraction, risk decision making, paired associate learning, visual search, sensorimotor speed, and vigilant attention (Basner et al., 2015). The battery has 15 unique stimulus sets that allow for repeated administration; it has French, Italian, German, and Russian translations; and factors that allow for correction of practice and stimulus set effects (that can otherwise confound the effects of interest) have been previously established (Basner et al., 2020a).
• Spatial Cognition—This evaluation includes a short battery of performance measures specifically targeting key spatial abilities including spatial updating (Wolbers et al., 2008; Stahn et al., 2020), path integration (Goeke et al., 2013), topographic mapping (Hartley and Harlow, 2012; Moodley et al., 2015), and visuo-spatial memory in small scale spaces (Riecke et al., 2010; Nguyen-Vo et al., 2021). The battery relies on well-validated paradigms, has been tested in various spaceflight analogs including bed rest, isolation studies, Antarctica, and parabolic flight, and has been flight-certified by NASA for use on ISS.
• ROBoT-r—The assessment simulates Canadarm2 track-and-capture activities. The task requires use of dual hand-controllers (6 degrees of freedom) to grapple an incoming vehicle in free-drift in a time-limited setting (Ivkovic et al., 2019).

In the Standard Measures protocol tasks are administered at least twice pre-flight, once early in-flight, once mid in-flight, once late in-flight, and twice post-flight. While we acknowledge restrictions in available crew time, we recommend increasing the administration frequency to better characterize each astronaut and the associated performance trajectory during all phases of a mission/study. The pre- and postflight performance assays should be performed on the same day, or as near to the time of the MRI scan as possible, to support the interpretation of the functional and structural imaging data (see also Section 2 Health surveillance neuroimaging recommendations).

#### Sensorimotor measures

Expanded standardized sensorimotor measures should include not only posturography but also spatial orientation, field tests, and unimanual and bimanual coordination tests.

The negative effects of spaceflight on sensorimotor function are well-documented. These include post-flight impairments in posture control (Layne et al., 2001; Cohen et al., 2012) and locomotion (Bloomberg and Mulavara, 2003; Miller et al., 2010; Mulavara et al., 2010; Cohen et al., 2012), as well as reduced mass discrimination abilities (i.e., reduced ability to identify differences in the mass of two different objects) (Ross et al., 1984) and decreased otolith function to detect gravity (Hallgren et al., 2016), leading to a decreased vestibulo-autonomic reflex (Hallgren et al., 2015). Additionally, negative effects of spaceflight on behavior include in-flight spatial disorientation and dizziness (Young et al., 1984) and changes in gaze holding in response to altered gravity (Clément et al., 1993). Astronauts in microgravity also encounter changes in the perception of self-motion; for instance, one study found immediate alterations in one’s perception of self-motion in the upwards/downwards (pitch), but not left/right (yaw) directions through a virtual tunnel when free-floating in weightlessness, suggesting that weightlessness may negatively affect the early processing stages of self-motion (i.e., vestibular and optokinetic function; see De Saedeleer et al. (2013). Likewise, data collected during parabolic flights have shown that the ability to continuously form and update transient sensorimotor representations about self-to-object relations during locomotion can be impaired during altered gravity levels (Stahn et al., 2020).

Because these behaviors all have high operational relevance, we recommend measurement of manual function, posture control and eye-head coordination. The impact of brain and ocular changes with spaceflight on these metrics is yet to be established, but associations between brain structural changes and these behaviors in spaceflight and bed rest in small samples support that further investigation is warranted (Lee et al., 2019a,b; Roberts et al., 2019). We recommend that these measures include posturography (as currently implemented), tests of spatial orientation (rod and frame, line orientation matching, or similar), spatial updating, path integration, and both unimanual and bimanual coordination (assessed via pegboard or similar tests). Test dates should be coupled with MRI scanning to aid in brain-behavior change interpretations. Pre, in and postflight testing should be included, with field tests administered as close to landing as possible to best understand changes in function.

### Recommendation level 1–5: Identify relevant contributing factors

Clarify potentially contributing factors to structural brain and eye changes such as genetics, sex, age, body mass and body mass index, exercise, sleep, environmental exposures, diet, etc.

Microgravity seems to be the key contributing factor for developing brain changes and SANS, as these changes normally do not occur on Earth. Susceptibility varies by individual, however, since within the same mission, only some astronauts are affected. The science community can potentially identify the source(s) for that variability by examining interindividual and environmental factors that might influence and contribute to the observed etiology. Identifying predictors of brain, behavior and ocular changes can lead to better mechanistic understanding and also potentially lead to identification of individual crewmembers needing additional monitoring, training or countermeasures.

#### Definition of spaceflight-associated neuro-ocular syndrome

The definition of former VIIP, now SANS, has changed throughout the years, which made the comparison of affected cases difficult. The latest definition for the presence of SANS is one or more signs in one or both eyes of chorioretinal folds, change in cycloplegic refraction > +0.75 diopters, globe flattening and a peripapillary increase of total retinal thickness > 20 μm. Applying this definition, about 68% of NASA crew members have been affected by SANS with a predominance of the right eye, which led to the hypothesis that some left-right anatomical difference might play a role in SANS development. Importantly, with increasing mission length and mission quantity, the incidence of SANS is increasing.

#### Influence of microgravity and fluid shift on different upper body pressure values

##### Intracranial and intraocular pressure

Due to globe flattening, choroidal folds, protruding optic nerve head, and increase in ventricle volume in SANS, researchers hypothesize that microgravity causes a disproportionate elevation in ICP relative to intraocular pressure (IOP) and thus reduction in the translaminar pressure gradient (TLPG) across the posterior part of the eye (the lamina cribrosa separates the globe from the subarachnoidal space and the TLPG is defined as IOP minus ICP.) Potentially contrary to this simple hypothesis, experimental data from parabolic flight found no increase in ICP, but rather a decrease compared to supine posture (Lawley et al., 2017). It was hypothesized that ICP in weightlessness will stabilize at values between that of upright and supine posture on Earth (Eklund et al., 2016). Moreover, ICP did not increase progressively during 24 h of head-down tilt bed rest. We only see indirect signs of an elevated ICP in postflight MRIs but no standard measurement of ICP inflight has been performed so far. The initial hypothesis that ICP would be—by clinical ground-based standards—pathologically elevated in space is thus not directly supported. The pathology of SANS may be due to the lack of habitual variability of both ICP and volume associated with posture changes on Earth, which may lead to a low, but consistent overload of cerebral structures and the noted structural remodeling.

##### Optic nerve fluids

Microgravity leads to optic nerve swelling due to excessive interstitial fluid accumulation in the tissues. Thus, the removal of fluid overload in the optic nerve remains a challenge. Unlike the retina and optic nerve, the optic nerve head doesn’t have a specific blood-tissue barrier (Tso et al., 1975). The lack of a specific barrier may predispose the optic nerve head to fluid accumulation during the cephalad fluid shift. Other possible sources of entry of excess fluid in the optic nerve head also exist. They include fluid streaming from tissues surrounding the optic nerve such as optic nerve glymphatics, the optic nerve capillaries, peripapillary choroid, vitreous, and CSF (Mathieu et al., 2017). The drainage of the lymphatic and glymphatic system depends also on the vascular stasis in the upper body, as well as the increased jugular vein pressure (Zhang and Hargens, 2018; Marshall-Goebel et al., 2019). The pulsation of the intracranial vessels as well as the lack of the different gravitational body positions with a change in pressure and fluid circulation seem to have an influence on fluid pooling. The IOP also influences the retinal nerve fiber layer, the prelaminar region of the optic nerve, and lamina cribrosa. A high intraocular pressure can lead to a reduction of perfusion, a reduction in nerve fiber layer thickness and a bulge of the lamina cribrosa in the posterior direction to the nerve. The lymphatic and brain-specific glymphatic circulation have recently been established as a fluid removal system for both the eye (Yücel et al., 2009) and the CNS and might also be impaired in microgravity. Recent approaches to study glymphatic clearance in humans could be applied postflight to better understand how microgravity impacts this system.

#### Given this background, the following factors may contribute to spaceflight-associated neuro-ocular syndrome and should be further investigated

##### Age

Most astronauts are at least in their mid-forties when they spend 6 months in space, and many of them have already experienced a previous spaceflight. Thus, whether older astronauts are more affected than younger ones is difficult to determine. Increasing vessel changes with age, additionally induced by space radiation, may play a role. Incidences of SANS and brain changes should be examined as a function of age and spaceflight experience.

Venous sinus congestion: There is a redistribution of venous blood volume toward the head with spaceflight (Hargens and Richardson, 2009; Hamilton et al., 2012; Marshall-Goebel et al., 2019). A small sample of astronauts who developed SANS inflight exhibited greater volumetric increases in the dural venous sinus volumes from pre to postflight than those that did not develop SANS (Rosenberg et al., 2021). Herefore, anthropomorphic factors like body weight might also play a substantial role in the triggering of SANS (Buckey et al., 2018). Thus, this should be further investigated as a potential mechanism to explain the individual variability in SANS development.

##### Advanced resistive exercise device as countermeasure

The advanced resistive exercise device (ARED) is used as a countermeasure against muscle and bone loss. Unfortunately, it may potentially increase intrathoracic pressure during valsalva-like behavior whilst thereby increasing ICP and potentially fostering the evolution of SANS. This hypothesis is anecdotally supported by the lower number of observed SANS cases in cosmonauts, who seem to use the ARED less often and at lower strength regimes than the USOS crew. A recent study shows that the perivascular space in the brain is significantly increased in the NASA crew with 25.5%, compared to Roscosmos crew with only 12.4% (Barisano et al., 2022). As all crews experienced the same environment aboard the ISS, the authors hypothesize that the differences in PVS enlargement may have been due to, among other factors, differences in the use of ARED and LBNP, which can influence brain fluid redistribution. Laurie and collaborators showed that the use of LBNP in-flight reduces intraocular pressure, supporting the potential effectiveness of this countermeasure against headward fluid shift during spaceflight (Greenwald et al., 2021). Moreover, as shown by Barisano et al. (2022), NASA astronauts who developed SANS already had greater pre- and postflight PVS volumes than those unaffected. Hence, these results provide evidence for a potential link between PVS fluid and SANS.

ARED is a very important device to keep muscle and bone mass during spaceflight. The potential negative effects on SANS should be evaluated in analog studies with ARED-like devices that increase the intrathoracic pressure.

##### Sex

When eye changes were detected in 2008, the hypothesis formed that women are less affected than men. This possibility is difficult to prove with available data since women have so far represented only about 11% of all space flyers. Brain changes have been investigated also in Russian astronauts which have been mainly men in the last decades. Therefore, not much data on female astronauts or cosmonauts exist to draw robust conclusions.

##### Genetics

Zwart and Smith discovered in 2012 that astronauts with refractive changes more often have a higher preflight serum homocysteine and cystathionine concentration. Higher preflight serum concentrations of 2-methylcitric acid also tended to be associated with ophthalmic changes. These biochemical differences suggest that the folate- and vitamin B-12-dependent 1-carbon transfer metabolism was affected before and during flight, and that polymorphisms in enzymes of this pathway may interact with microgravity. These differences existed already before flight, suggesting that they may contribute to SANS risk (Zwart et al., 2012). This relationship should be investigated in future astronauts and bed rest test subjects.

##### Nutrition

Several studies suggest that consuming food rich in antioxidants and anti-inflammatory components such as those found in fruits, nuts, vegetables, and fish may reduce age-related cognitive decline and the risk of developing various neurodegenerative diseases (Melzer et al., 2021). Food that is rich in omega-3 fatty acids is garnering appreciation for supporting cognitive processes in humans and upregulating genes that are important for maintaining synaptic function and plasticity in rodents (Gómez-Pinilla, 2008). It even decreases inflammation, cellular death, and damage to the axons in rodent models after traumatic brain injury which might support the regeneration of brain changes during spaceflight (Smith-Ryan et al., 2020). Adequate food supply to keep a healthy level of vitamins (e.g., Vitamin B 6, 12, A, C, E), antioxidants and omega-3 fatty acids is important to reduce additional risk factors for eye and brain changes and will be a challenge for future very long space missions. Food intake by USOS crew and Russian crew is, in practice, very similar due to exchange of the meals (personal communication). Hence, the difference between NASA and Roscosmos crew regarding the incidence of SANS should not be attributed to the diet.

##### Sleep deprivation

Sleep deprivation is prevalent in spaceflight (Barger et al., 2014). It not only affects cognitive performance and metabolic health (Banks and Dinges, 2007), but also is a potent risk factor for the development of neurodegenerative disease (Havekes et al., 2019). For these reasons, insufficient sleep is likely an important contributing factor to spaceflight induced changes in brain structure and function. Polysomnography, i.e., the simultaneous measurement of the electroencephalogram, electromyogram and electrooculogram, is the gold standard for measuring the most important aspects of sleep. However, application of electrodes is cumbersome, and the instrumentation is somewhat invasive and may influence sleep, which is why to date few polysomnographic studies have been performed in space (Basner and Dinges, 2014). New and easier to handle measurement devices (e.g., Arnal et al., 2020) have recently become available that will facilitate polysomnographic measurements in spaceflight and space analog environments. Obtaining this type of sleep data is particularly important given that CSF turnover and glymphatic clearance are both enhanced during sleep. Understanding sleep disturbances during spaceflight may thus lead to new approaches for enhancing these processes.

#### Potential contributing factors of spaceflight-associated neuro-ocular syndrome that had been previously investigated

##### Salt

Astronaut food was previously high in salt to make it tastier. High salt intake could be a possible contributing factor for fluid retention and swelling of the head mucosa; therefore, salt intake has been reduced in the astronauts’ food. This reduction did not subsequently reduce SANS development.

##### Carbon dioxide

High CO_2_ on the space station has been suggested as the cause for eye changes, because of potential vein vasodilation and the resulting increased cerebral blood flow. To explore this possibility, an :envihab research facility head down tilt bed rest study aimed to simulate the effects of microgravity on the human body under increased CO_2_ conditions. Test subjects were exposed to 0.5% CO_2_ for 30 days during the bed rest phase. During this study, called VaPER (VIIP and Psychological :envihab Research Study), optic disc edema was present in 5 out of 11 test persons. This study had applied a strict head down tilt with no pillow use (Laurie et al., 2019) and increased CO_2_ levels. It was not clear which of these variables were responsible for the first-time detection of optic disc edema on Earth.

The AGBRESA (Artificial Gravity Bed Rest—European Space Agency) study also used a strict head down tilt position for an even longer time period (60 days), but with an ambient air environment. During this study, optic disc edema was also detected in test persons under normal CO_2_ conditions. Thus, even if the bed rest analog is not a complete simulation of space flight effects, elevated CO_2_ levels comparable to that found on ISS do not seem to play a major role in the development of SANS (Laurie et al., 2021). While Earth-based analogs of the spaceflight environment certainly have their limitations and do not replicate all aspects of microgravity, the fact that signs of SANS have been observed in this environment make it a reasonable approach to studying mechanisms and potential countermeasures. However, head down tilt bed rest is not a perfect match to all features of human spaceflight. One of the main problems may be that −6 degree is not sufficient to fully model SANS, but more than −10 degrees of head tilt is not tolerable for most subjects over longer periods of time and might be unethical due to long-term ocular and retro-orbital tissue alterations. This is a dilemma with no clear solution and full validation of countermeasures will probably only be achieved in the real-life mission scenarios aboard the ISS.

#### Contributing factors recommendations

We recommend that all the following factors be measured and evaluated in relation to SANS to determine whether they contribute: peak CO_2_ exposure during a mission, ARED use, lower body negative pressure (LBNP—see section “recommendation level 1–6”) use (frequency and intensity), age, sex, folate status, one-carbon biochemistry, sleep quality and quantity, crewmember body mass, body mass index, salt intake, radiation exposure, frequency of prior spaceflights, total number and duration of prior spaceflights. Many of these factors could be examined using existing datasets via data mining.

### Recommendation level—1–6: Study countermeasures’ impact on sans and brain

Investigate a targeted portfolio of exercise and other countermeasures including dosage strategies.

Currently, there are no SANS-specific countermeasures implemented for routine use in-flight. Exercise and LBNP are already in use on the ISS and should be further developed and evaluated. Several mechanical, nutritional, and pharmacological countermeasures, as well as sensory stimulation, are under investigation, while correlations to aggravating factors such as resistive exercise are under review. Adequate countermeasures to prevent or cure SANS are important on an operational level to support astronauts. They also play a key role in understanding the underlying mechanisms and pathologies of this syndrome. Current research is focused on countermeasures for 0 G; in future discussions, the effects of the partial gravity environments of the Moon and Mars may play an important role.

Because headward fluid shift seems to be a key factor in initiating or aggravating SANS, efforts have been directed toward reversing this fluid shift and thereby unloading cerebral structures. A few approaches are recommended below.

#### Lower body negative pressure

Multiple in-flight and ground-based experiments have, or are currently, investigating efficacy of various mechanical countermeasures on a proof-of-concept level (minutes) or on a short-term level (a few days or 1–2 months using bedrest) to reverse the fluid shift. Lower body negative pressure (LBNP) was suggested as an integrative countermeasure as early as the 1970s (Gurovsky et al., 1973) and has been implemented in-flight during SpaceLab, MIR, and ISS (Charles and Lathers, 1994). This technique has a long history of application (inflight and in ground analogs) and has been shown to unload cerebral structures following a non-linear dose-response relationship. It might also reduce ICP which has not been directly measured inflight so far, but astronauts do report a favorable unloading of the brain feeling. It reduces IOP but not choroidal thickness in astronauts aboard the ISS (Greenwald et al., 2021). Cosmonauts using LBNP inflight also seem to show less SANS. Using LBNP during the whole mission might be effective not only in reducing SANS, but also in reducing the possibility of venous thrombosis, by potentially restoring normal venous hemodynamics and in maintaining blood vessel function and reflexes (Arbeille et al., 2012, 2021; Lee et al., 2014). In 12°HDT with -20 mmHg, LBNP was able to reduce the increase in optic nerve sheath diameter, which is linked to ICP and intracranial CSF (Marshall-Goebel et al., 2017). LBNP was applied as a countermeasure for 6 h per day during the recent NASA study at :envihab (DLR). A recent randomized crossover trial for LBNP in a model of SANS showed the effectiveness of 8 h of LBNP on the choroid engorgement, supporting the growing evidence for LBNP as an effective countermeasure for SANS (Hearon et al., 2022).

The best parameters for applying LBNP still need to be determined. Questions concerning the time (day or night), length, frequency, and amount of pressure need to be answered. The feasibility of long-term LBNP use is also under consideration. Finding the correct pressure is difficult too because of safety concerns related to the cardiovascular response, with potential syncope and reduced central perfusion pressure. The recent MRI based study by Kramer et al. (2022), showed that 25 mmHg of LBNP did not decrease cerebral perfusion, neither internal jugular vein flow stasis. Indeed, 20–25 mmHg seems to be an adequate pressure (Harris et al., 2020), and studies performed 20 years ago showed an advantage for individually determined pressure values (personal communication, Juergen Drescher). Also, whether LBNP should be applied to “prevent or cure” has not been answered yet. More studies in-flight and in ground analogs using LBNP with individual thresholds should be performed with longer usage, preferably during the night, and with newly developed LBNP systems that allow astronauts to sleep and work while in the apparatus.

#### Artificial gravity

Artificial gravity has been discussed for many years as the best potential countermeasure against SANS (among other physiological systems that would also benefit) on long duration flights. Physicians hope that artificial gravity (AG) could dramatically reduce the adverse effects of microgravity, but engineers have not yet successfully created a system for use in microgravity. During the Neurolab Shuttle mission in 1998, four astronauts were subjected to AG in-flight on the centrifuge called the Visual Vestibular Integration System (VVIS). After flight, their vestibular function was not decreased nor did they show signs of orthostatic intolerance, unlike their counterparts who did not undergo AG (Moore et al., 2001).

Many questions concerning the effects of intermittent gravity on the vestibular system (including spatial disorientation, vertigo, and motion sickness) and on the adaptation to different partial gravity levels (Moon and Mars) still remain and could be explored further in low Earth orbit and ground-based studies. The application of AG during bed rest studies (such as AGBRESA) has not proven to be sufficient for preventing SANS, which might be attributable to the intensity and length of the AG. More studies, with higher AG levels administered for longer durations such as planned during the next ESA Head Down Bed Rest campaigns (2025–2026), should be performed to answer the general question of whether application of AG affects the development of SANS or undesirable effects on the vestibular system and cognition.

#### Exercise

An extensive body of research suggests that participation in regular exercise programs goes well beyond improving cardiovascular and musculoskeletal fitness, by affecting brain health, behavior, and mental wellbeing. Systematic reviews and meta-analytic studies have reported robust relationships between higher fitness levels and physical activity and brain gray matter volume (Erickson et al., 2014), white matter volume (Sexton et al., 2016), and functional brain network connectivity (Chen et al., 2020; Bray et al., 2021; Domingos et al., 2021; Yu et al., 2021). These findings have been observed across the life-span (Voss et al., 2011; Heinze et al., 2021), and reveal consistent effects in brain regions including, but not limited to, the frontal lobes (prefrontal and temporal cortices), anterior cingulate cortex (ACC), and the hippocampus (Erickson et al., 2014; Firth et al., 2018; Domingos et al., 2021; Ji et al., 2021). These areas are critically important for higher cognitive functions, such as multidimensional executive and control processes, learning, and memory formation. Collectively, these data suggest that exercise could also be a potential countermeasure to mitigate adverse neurobehavioral effects associated with long-duration spaceflight. This suggestion is supported by recent evidence from bed rest studies using task functional imaging (Friedl-Werner et al., 2020). Likewise, bed rest data also confirm the potential of exercise as a cue for maintaining circadian rhythm (Mendt et al., 2021), which is also linked to optimal cognitive performance (Chellappa et al., 2018).

The ability of certain types of exercise, frequency, intensity, and duration to maximize such brain effects is still unclear, and the best exercise program for the brain may be different from protocols optimized to minimize bone and muscle loss, or cardiovascular deconditioning. The best effects may be achieved by combining physical exercise with a sensory stimulation that immerses the user in a virtual 3D environment (Vessel and Russo, 2015). Such a multi-countermeasure approach could deliver an increased level of experiential diversity, which has been shown to be critical for brain plasticity (Stahn et al., 2019; Heller et al., 2020). However, there are also suggestions that resistive exercise may increase intraocular and ICP, which should be taken into consideration when using exercise as a potential countermeasure (Zhang and Hargens, 2014; McMonnies, 2016).

#### Nutrition

As alluded to previously, Zwart et al. (2016) have shown that B vitamin status is a significant predictor of SANS, suggesting that a simple nutritional countermeasure may at least partially reduce the risk of SANS.

## Level 2 priority recommendations

### Recommendation level 2–1: Increase the number of female astronauts

Increase statistical power for sex comparisons.

It is currently unclear from available published data whether women and men show similar brain and ocular changes with spaceflight, since only about 11% of space travelers have been women (Smith et al., 2020). In head down tilt bed rest studies, no sex difference in developing optic disc edema could be detected (Laurie et al., 2019). For a better understanding of potential sex-related factors considering this asymmetric representation, we need better access to past and future research data to achieve robust calculations. This recommendation is also in line with the NIH 2015 policy mandate to investigate sex as a biological variable^1^.

In addition, an extensive body of research demonstrates significant sex-dependent molecular, structural and functional brain differences (e.g., Cahill, 2014). Recent large-scale and meta-analytic studies reveal structural and functional brain differences between men and women, including brain gray and white matter volumes and structures critically involved in spatial navigation such as the hippocampus, caudate nucleus, and precuneus (Ritchie et al., 2018).

On Earth, males also typically outperform females on motor and spatial cognitive tasks, while females are faster in tasks of emotion identification and non-verbal reasoning (Satterthwaite et al., 2015). Further, spatial ability and its neural circuitry show sex-specific differences. Considerable evidence exists for sex-dependent differences in spatial navigation performance and strategies (Boone et al., 2018), which have been associated with different levels of hippocampal activation (Grön et al., 2000). It is unclear whether such differences also exist in astronauts, and if and to what extent, spatial navigation and related brain changes are differently affected after spaceflight in men and women. Sex differences have not been reported in brain changes with flight to date. Reschke et al. (2014) found that female astronauts more frequently reported space motion sickness and postflight vestibular instability symptoms than males, but objective, quantitative postflight postural control measures show no difference in occurrence.

Visuo-spatial abilities are involved in various operational skills such as docking, landing, exploring, and navigating in new environments and on planets with low gravity. Spaceflight may be associated with potential adverse visuo-spatial effects. While navigating on other planets, astronauts will lack familiar landmarks, have a loss of aerial perspective, and an altered sense of balance caused by ambiguous depth cues during altered gravity. In addition, the whole brain including regions critical for spatial memory and learning may be affected during prolonged exploratory class missions as a result of cosmic radiation (Kiffer et al., 2019), hypercapnia (Kiray et al., 2014), or isolation and confinement (Stahn et al., 2019). Given the pivotal role of spatial cognition for human spaceflight operations, elucidating the effect of space and any sex-related brain plasticity, behavior and the molecular mechanisms is crucial. This notion is in line with the priorities of many of the world’s leading research centers to consider sex as a key biological variable (Clayton, 2018), and the space agencies’ goal to increasingly strengthen the role of women in future space expeditions. Due to the increasing and essential role of women in future space missions, studies of both men and women are critically needed to better understand sex-dependent vulnerabilities or resilience to adverse neurobehavioral effects during future exploratory space expeditions such as a mission to Mars.

### Recommendation level 2–2: Increase research in space analog environments

Conduct research to investigate whether eye and brain alterations occurring in space analog environments are similar in magnitude and underlying mechanisms to the actual spaceflight-induced structural alterations.

Future exploration space expeditions will push the limits of human performance. Crew members will face hostile environmental conditions, high operational demands, and significant psychological stressors. To ensure safe and successful human space exploration, understanding the nature and interactions of these stressors is critical. This knowledge will allow for predictions about potential adverse effects on brain and behavior during exploration class missions that go well beyond current standard missions on the ISS. Most operational and environmental stressors can be simulated in spaceflight analogs. Two analogs that deserve particular consideration are bed rest and isolation studies using isolated and controlled confinement (ICC) and isolated, confined and extreme (ICE) (Choukér and Stahn, 2020).

Bed rest and dry immersion provide excellent models to mimic some of the neurophysiological adaptations associated with body unloading. An increasing body of research pinpoints the criticality of physical activity for brain plasticity across the lifespan (Voss et al., 2019). The effects of regular exercise are particularly evident for hippocampal plasticity and spatial learning (Erickson et al., 2011). Conversely, body unloading and reduced physical activity levels during space missions could be a significant risk factor for brain health, cognitive performance, and mental wellbeing. Long-duration bed rest has been shown to induce an upward shift and posterior rotation of the brain, widespread morphologic changes with brain tissue redistribution and intracranial fluid redistribution (Roberts et al., 2015; Koppelmans et al., 2017b), and effects on sensorimotor control, vestibular function and functional mobility (Koppelmans et al., 2015, 2017a; Yuan et al., 2018), selective attention (Brauns et al., 2021), affective processing (Brauns et al., 2019; Basner et al., 2021), neural efficiency of memory encoding and retrieval (Friedl-Werner et al., 2020), dual-tasking (Yuan et al., 2016), and spatial working memory (Salazar et al., 2020). Dry immersion also mimics the unloading and reduced physical activity levels of spaceflight. It remains unclear whether it is an effective analog for brain and ocular changes as well, but current ongoing studies are exploring this possibility.

Isolation and confinement and its associated stressors could also potentially impact neuroplasticity and cognitive performance during long duration space missions. Reduced sensory stimulation and resulting sensory monotony associated with such missions pose a serious unmitigated risk for adverse behavioral conditions and psychiatric disorders (Vessel and Russo, 2015). The absence of privacy, constant interaction within the same group, and little separation between work and leisure, as well as constant sleep/work shifts and sleep disruptions onboard, create potential independent stressors that may interact with each other (Palinkas, 2001).

Various lines of research show that social isolation can increase sympathetic tone, oxidative stress, and the expression of genes regulating glucocorticoid responses, ultimately leading to glucocorticoid resistance (Cacioppo et al., 2011). Due to its high density of corticosteroid receptors, the hippocampus and particularly the dentate gyrus are highly vulnerable to increased stress hormone levels (Fa et al., 2014). Animal studies have shown that social isolation reduces neurogenesis, long-term potentiation, and key neurotrophins in the hippocampus, and worsens memory performance (Scaccianoce et al., 2006; Cinini et al., 2014; Kamal et al., 2014). Neuroimaging, biochemical, and behavioral data from Antarctic expeditioners show that prolonged social isolation and confinement reduces gray matter in the prefrontal cortex, hippocampus, and parahippocampus (Stahn et al., 2019). Further, the Antarctic participants showed gradual decreases in key neurotrophins and flattened learning trajectories of cognitive tasks associated with spatial abilities, selective attention, and response inhibition. Likewise, preliminary evidence suggests that short periods of isolation and confinement, and particularly in combination with additional stressors such as sleep deprivation, can impair brain plasticity and complex cognitive abilities such as spatial navigation.

Together, these data highlight the significance and value of bed rest, ICCs, and ICEs as analogs to systematically study and better understand the role of distinct environmental, operational, and psychological stressors associated with prolonged spaceflight and to develop target-specific countermeasures to mitigate their risks. These analogs will continue to be critical platforms to ensure safe and successful human spaceflight missions.

### Recommendations level 2–3 establish easily accessible database of ocular/magnetic resonance imaging/behavioral data and easily accessible database of supplemental data

– Build a data archive of de-identified, internationally sourced data (NASA, ESA, Roscosmos, Chinese Space Agency, JAXA, CSA, etc.) from past government-funded experiments with low-threshold structured access to allow for broader retrospective research.
– Mandate the upload of RAW and unprocessed but de-identified experimental data using open and accepted international data formats to avoid vendorexclusive data solutions.
– Provide access after completion of the experiment to interested researchers for additional analysis, including recognition of the original PIs.
– Assure the general accessibility of relevant supplementary in-flight data [CO2, sleep hours, activity data, exercise programs, medication logs, countermeasure logs, biochemical data, nutrition, accumulated radiation exposure, number and duration of EVA’s, and medical issues (space motion sickness, headaches, back pain, etc.)].

To advance, maximize, and speed up scientific discoveries, we advocate the use of data repositories for core neuroimaging, ocular, behavioral and medical data. These archives should go beyond existing platforms by (1) promoting data transfer between agencies wherever possible, (2) supporting efficient data access with respect to global IRB and data privacy laws and regulations, (3) establishing universal and internationally accepted data formats for all measures, (4) providing processed and unprocessed data, and (5) supporting the integration with data collected by individual experiments, i.e., outside the proposed standard measures where needed.

In many cases, important data are collected either for research purposes or for clinical monitoring and are not made immediately available to test for cross associations. Many features such as sleep hours, CO_2_ levels, exercise routines, nutrition, supplements, accumulated radiation exposure, number and duration of EVA’s, medication, etc. are likely to interact with spaceflight-induced brain and ocular changes. These data need to be made available to clinicians and researchers in an accessible and timely manner. We are aware that NASA’s data repositories have more information than the European data repositories. Historically, the NASA system has had a slow approval and transfer process. This fact greatly impedes progress in obtaining a holistic understanding of the factors that may increase risk or be antecedents for brain and ocular changes with spaceflight. We know that the NASA data system is currently being revised to accommodate new types of data digitally and to facilitate ease of access, data upload, and analytics. We hope that the developers will consider our recommendations during their revisions.

The team certainly understands the need for health and medical privacy of crewmembers. Increased transfer of data and knowledge between medical operations and scientists is required, however, to make the best progress toward understanding brain and ocular changes. Moreover, international data sharing also needs to be better streamlined and facilitated. The current processes are cumbersome and not well understood within the community.

## Conclusion

The expert panel concludes that there are several avenues of research that should be initiated (and in some cases, that have been initiated, but should receive continued attention) to better understand human brain, ocular, and behavioral changes with spaceflight. The findings to date provide strong evidence that these changes already have operational impact and may influence long-term health. To understand and mitigate these effects, we recommend better delineation of flight duration dose-response effects, missions longer than 1 year on the ISS, investigation of partial-g effects and transitions, institution of state-of-the-art neuroimaging capabilities, an emphasis on the evaluation of integrative countermeasures and evaluation of sex differences. Furthermore, enabling streamlined access to international data sets across space agencies would allow for more rapid and potentially transformative insights.

We refer the reader to the Appendices following the references for additional information about medium and lower priority recommendations, suggested MRI protocols and core OCT protocols.

## Data availability statement

The original contributions presented in this study are included in the article/supplementary material, further inquiries can be directed to the corresponding author.

## Author contributions

MB analyzed the paired comparison and created the figure. All authors listed have made a substantial, direct, and intellectual contribution to the work, and approved it for publication.

## Footnotes

1
https://orwh.od.nih.gov/sex-gender/nih-policy-sex-biological-variable

## Abbreviations

AG: Artificial Gravity
AGBRESA: Artificial Gravity Bed Rest—European Space Agency
ARED: Advanced Resistive Exercise Device
ANAM: Automated Neuropsychological Assessment Metrics
CNS: Central nervous system
CO_2_: Carbon dioxide
CSA: Canadian Space Agency
CSF: Cerebrospinal Fluid
CSFP: Cerebrospinal Fluid Pressure
ESA: European Space Agency
EVA: Extravehicular Activity
g: Acceleration due to gravity
ICC: Isolated and Controlled Confinement
ICE: Isolated extreme Environment
ICP: Intracranial Pressure
IOP: Intraocular Pressure
ISS: International Space Station
JAXA: Japanese Space Agencies
LBNP: Lower Body Negative Pressure
LEO: Low Earth Orbit
MRI: Magnetic Resonance Imaging
NASA: National Aeronautics and Space Administration
OCT: Optical Coherence Tomography
PC: Paired Comparison
PET-MRI: Positron Emission Tomography–Magnetic Resonance Imaging
PI: Principal Investigator
QC: Quality Control
R: Return
ROBoT: Robotic On-board Trainer
SANS: Spaceflight-Associated Neuro-ocular Syndrome
TLP: Translaminar Pressure
TLPG: Translaminar Pressure Gradient
UNO: United Nations
USOS: United States Orbital Segment
UTMB: University of Texas Medical Branch
VaPER: VIIP and Psychological :envihab Research Study
VIIP: Visual Impairment and Intracranial Pressure
VVIS: Visual Vestibular Integration System
WHO: World Health Organization
WinSCAT: Windows Spaceflight Neuro-Cognitive Assessment Tool.

## Appendix

### Appendix A: Additional medium and low priority topics not described in the main text

#### Recommendation level 2–3: Increase interdisciplinary research

##### Advocate for interdisciplinary eye and brain research

Most of the space-related research (with the laudable exception of a few investigators) still treats SANS (ocular) research and the brain structural alterations as separate entities; however, a common mechanism related to a cephalad fluid shift driving both phenomena is highly likely. A shift toward more interdisciplinary research projects is needed, including at minimum specialists and studies from ophthalmology, neurology and neuroradiology. A predictive biomarker for the effects of cephalad fluid shift is more likely to be generated from a combination of parameters across disciplines rather than from one specialty alone. Interdisciplinary research is likely to accelerate scientific progress substantially.

#### Recommendation level 2–4: Promote interagency research

##### Drive interagency research projects to tackle urgent research projects faster by reaching statistical threshold earlier—Set-up an interagency research funding system

Consider creating a global research structure that can bond the efforts of all space agencies sending humans in space, to unify the research questions and pool the data obtained from the crew of different agencies. An interagency approach would better enable detailed monitoring of the brain and other crew health effects and performance of necessary data analysis.

#### Recommendation level 3–1: Spaceflight participant data

##### Let non-professional spaceflight participants (tourists) participate as much as possible in core data and research data acquisition to increase sample size and sample variability

Twelve space tourists have already flown to the ISS, with the first one being Dennis Tito in 2001. This group includes only one woman. Adding the data from this cohort in the science program would significantly increase the data pool and variance. Not only do spaceflight participants follow other routines onboard the ISS, their age range and physical fitness level differ from the other crew that are selected by space agencies. Also, applying different routines on board the ISS, this group increases the variability in the data set considerably, allowing for new findings. Hence, space tourists should be encouraged to participate maximally in the science programs of space agencies. With Space-X and other spaceflight providers, the number of space tourists will increase significantly.

#### Recommendation level 3–2: Promote autopsy in deceased astronauts

It is difficult to examine brain and eye changes histologically *in vivo*, as we do not have direct molecular access to all structures responding to space and microgravity. Therefore, examinations of the brain and eyes posthumously might give additional insight into the structural deteriorations and aging processes. These autopsies should be performed in cooperation with specialists who have knowledge about spaceflight-induced changes in the body.

## Appendix B: Proposed core MRI protocol

### Recommendation level 1–4: Standardized core measures for space and analog environments (additional details)

#### Health surveillance neuroimaging

**Measurements:** Keep **time of day** for scanning constant (4 h window) over within-subject measurements

**Scanner:** Any vendor 3T MRI with > >20 channel head coil and good head fixation system (e.g., memory foam) 3D sequences (for quantifiability and long-term applicability) should be used exclusively if applicable to the modality to guarantee maximal data quality (with respect to resolution and contrast-noise-ratio) and research usability in small cohorts as well as comparability for future studies. Standard T1 and T2-weighted 3D-images at 0.75–1 mm isotropic resolution in an up-to-date 3T scanner (minimum number of head coil elements 20) with a contrast-noise-ratio greater than 60 for any tissue type and a signal-to-noise-ratio greater than 20 reflect the bare minimum to detect morphological alterations to the brain. Sequences below are listed in order of weighted clinical relevance in case of subject measurement abort due to limited time or other factors. (Please note: all sequences are completely noninvasive; that is, no contrast agents are used, and no subject participation/interaction is needed):

1. 3D T1-weighted sequence (0.75 mm isotropic voxel size, FA 12°°, GRAPPA < <3) structural whole brain analysis (6:30 Min.) (eyes closed).
2. 3D T2-weighted sequence (0.75 mm isotropic voxel size, GRAPPA < <4) structural whole brain analysis including lesion detection, perivascular space quantification and myelin ratio in conjunction with the T1-weighted data of identical resolution (8:30 Min.) (eyes closed).
3. 3D Quantitative susceptibility map (1 mm, whole brain) e.g., for iron quantification (residues from thrombosis) and venous vasculature dilation measurement (6 Min.) (eyes closed).
4. 3D T2 SPACE ZOOMit (0.4–0.5 mm isotropic voxel size, TR 1,300 ms, TE 132 ms, fat suppression on) for eyeball and retro-orbital space lesion detection and optical nerve sheath assessment during eyes open with fixation straight ahead; slab can also be positioned/angled to concurrently evaluate the inner ear (10 Min).

Time permitting:

5.3D Time-of-Flight angiography (0.8 mm isotropic, FA20°°) arterial vasculature position, degree of elongation and tortuosity segmentation (8 Min.) (eyes closed).

6. T2-weighted high-resolution hippocampal sequence, at least 0.4 mm × 0.4 mm in plane resolution (5 Min.) in conjunction with T1-weighted sequence (#1) (see below for additional details).

7. Diffusion weighted sequence with isotropic voxels with at least 64 directions spread over multiple b-shells (e.g., B = = 0, 250, 500, 1,000, 1,500, 2,500 s/mm2).

(Total scanning time: core protocol 30 min full protocol 50 Min)

General tools for quality control (QC) in neuroimaging such as analysis for artifacts, contrast-to-noise-ratio, head motion artifacts, ghosting rate, Gibbs ringing with software such as MRIQC or the QC pipeline of the UK Biobank (including regular phantom measurements with the respective coil elements in each scanner) should be applied after the standardized data acquisition to ensure long-term usability (Esteban et al., 2017; Alfaro-Almagro et al., 2018). Data from different scanners and sites should be harmonized using empirical Bayesian approaches such as ComBat to account for technical variability (Beer et al., 2020).

#### Hippocampal imaging additional details

Hippocampal volumetry and morphometry have been employed to identify the presence and progression of various cognitive disorders. Similar types of imaging studies should be a part of space and analog testing as well. Aging, psychiatric disorders, and neurodegenerative diseases do not uniformly affect the hippocampus and its adjacent entorhinal, perirhinal, and parahippocampal cortices; some regions show a higher vulnerability than others (Adler et al., 2014). The hippocampus is arranged functionally along its longitudinal axis, and hippocampal subfields subserve distinct behavioral and cognitive functions (Strange et al., 2014). Typically, T1-weighted MRI scans at conventional resolution (at 3T magnet strength) cannot provide all the anatomical details needed to robustly identify the cytoarchitectonic differences that define the hippocampus (Yushkevich et al., 2015; Cong et al., 2018). Capitalizing on a uniform segmentation protocol proposed by an international expert group “Hippocampal Subfield Group (HSG)” (Wisse et al., 2017), we advocate the use of a harmonized T2-weighted sequence that will allow for investigators to robustly characterize normative and pathological hippocampal and parahippocampal changes in response to long-duration spaceflight. Imaging studies are particularly important because the low-dose irradiation expected during exploratory space missions can suppress hippocampal neuronal excitability, disrupt long-term potentiation, and elicit long-lasting effects in learning and memory formation (Acharya et al., 2019).

## Appendix C: Core ocular coherence tomography

The minimal recommended OCT routine is described below:

All: High resolution:

Circle (RNFL): ART 100 over disc

Radial: 20°, 24 sections ART 16, over disc

Disc: 20 × 20°°, 25 sections ARt 16

Line: 30°, EDI, ART 100-line through the center of fovea and disc

Vertical: 20 × 10°, sections 13, ART 16, between edge of disc and fovea
